# Mortality in Robin sequence: identification of risk factors

**DOI:** 10.1007/s00431-018-3111-4

**Published:** 2018-02-28

**Authors:** Robrecht J. H. Logjes, Maartje Haasnoot, Petra M. A. Lemmers, Mike F. A. Nicolaije, Marie-José H. van den Boogaard, Aebele B. Mink van der Molen, Corstiaan C. Breugem

**Affiliations:** 1 Department of Plastic and Reconstructive Surgery, University Medical Centre Utrecht, Wilhelmina Children’s Hospital, Utrecht, The Netherlands; 2 Department of Pediatrics, University Medical Centre Utrecht, Wilhelmina Children’s Hospital, Utrecht, The Netherlands; 3 Department of Clinical Genetics, University Medical Center Utrecht, Wilhelmina Children’s Hospital, Utrecht, The Netherlands

**Keywords:** Robin sequence, Pierre Robin sequence, Mortality, Congenital anomalies

## Abstract

Although Robin sequence (RS) is a well-known phenomenon, it is still associated with considerable morbidity and even mortality. The purposes of this study were to gain greater insight into the mortality rate and identify risk factors associated with mortality in RS. We retrospectively reviewed all RS infants followed at the Wilhelmina Children’s Hospital from 1995 to 2016. Outcome measurements were death and causes of death. The authors identified 103 consecutive RS infants with a median follow-up of 8.6 years (range 0.1–21.9 years). Ten of the 103 infants (10%) died at a median age of 0.8 years (range 0.1–5.9 years). Nine of these ten infants (90%) were diagnosed with an associated syndrome. Of these, seven infants died of respiratory insufficiency due to various causes (two related to upper airway obstruction). The other two syndromic RS infants died of arrhythmia due to hypernatremia and of West syndrome with status epilepticus. One isolated RS infant died of brain ischemia after MDO surgery. Cardiac anomalies were observed in 41% and neurological anomalies in 36%. The presence of a neurological anomaly was associated with a mortality rate of 40% versus 7% in infants with no neurological anomaly (*p* = 0.016), with an odds ratio of 8.3 (95% CI 1.4–49.0) for neurological anomaly versus no neurological anomaly. Mortality was 15% in infants with syndromic RS versus 2% in infants with isolated RS (*p* = 0.044). Mortality was not significantly associated with the presence of a cardiac anomaly, surgical treatment for severe respiratory distress in the neonatal period, or prematurity.

*Conclusion*: RS represents a heterogeneous patient population and is associated with a high level of underlying syndromes. The present study reports a mortality rate of 10% significantly associated with syndromic RS and the presence of neurological anomalies. A multidisciplinary approach in all infants born with RS, including genetic testing and examination of neurological anomalies in a standardized way, is crucial to identify infants with underlying syndromes potentially associated with increased mortality.

**Table:** 

**What is Known:**
• *Reported mortality rates in Robin sequence vary from 2% to 26%.* • *Clinicians mainly focus on the morbidity of Robin sequence that includes respiratory complications due to upper airway obstruction in the period after birth.* • *Robin sequence represents a heterogeneous patient population and is associated with a high level of underlying syndromes.*
**What is New:**
• *The present study reports a mortality rate of 10% significantly associated with syndromic Robin sequence and the presence of neurological anomalies.* • *A multidisciplinary approach in all infants born with Robin sequence, including genetic evaluation and standardized workup for neurological anomalies, is crucial to identify infants with underlying syndromes potentially associated with increased mortality.*

## Introduction

Robin sequence (RS) was first described by the French stomatologist Pierre Robin in 1923 and is characterized by the triad of micrognathia, subsequently leading to glossoptosis and varying degrees of upper airway obstruction [27]. RS is a congenital condition occurring in approximately 1 in 5600–8000 live births [24, 36]. Recently, an international consensus was achieved regarding the three distinguishing characteristics (micrognathia, glossoptosis, and upper airway obstruction) that should be included in the diagnosis of RS in newborns. Cleft palate is frequently encountered, but is not considered a prerequisite for the diagnosis [7]. RS infants represent a heterogeneous patient population because RS might be an isolated condition or be part of a syndrome (in about 26 to 83% of cases) [24, 29–31]. Clinicians mainly focus on the morbidities of RS, which include respiratory complications due to upper airway obstruction, feeding problems, a related failure to thrive, and the associated cleft palate problems, when present [10, 16, 33]. Reported mortality rates in RS vary from 2 to 26% [9, 11, 12, 14, 15, 19, 21, 29, 32, 33, 35, 37]. Upper airway management plays a central role in the treatment of RS. Treatment of the tongue-based respiratory obstruction minimizes the risk of hypoxic cerebral injury and repeated (aspiration) pneumonia [13, 18, 25]. Nonsurgical interventions include positional change, the nasopharyngeal airway, continuous positive airway pressure, and the palatal plate [1, 2, 16, 22]. However, when facing severe respiratory distress, surgical procedures are applicable, such as mandibular distraction osteogenesis (MDO), tongue-lip adhesion (TLA), subperiosteal release of the floor of the mouth, and tracheotomy [4, 6, 8, 17].

Limited information is available in the literature concerning the mortality in RS. Recently, Costa et al. demonstrated that mortality in RS is not always directly related to tongue-based respiratory obstruction. Cardiac and neurological anomalies were found to be associated with significantly increased mortality [12]. A better understanding of the mortality in RS and its relationship with cardiac and neurological anomalies might improve the multidisciplinary treatment of this complex congenital disorder.

The primary aim of this study was to gain greater insight into the mortality rate and characteristics of the deceased RS infants. The secondary aims were to identify the associated cardiac and neurological anomalies in RS and to identify factors potentially associated with an increased mortality in RS infants.

## Material and methods

In this retrospective cohort study, we included all infants that were admitted to the Wilhelmina Children’s Hospital and diagnosed with RS from 1995 to 2016. The study was approved by the medical ethical board (13-557/C). RS was defined as micrognathia, glossoptosis, and upper airway obstruction, with or without the presence of cleft palate. The Dutch Cleft Registry, managed by the Dutch Association for Cleft Palate and Craniofacial Anomalies, was used for patient identification and supplemented with information for infants that underwent surgery related to RS. Medical records of all RS infants were reviewed and analyzed in January 2017.

Patient characteristics that were obtained included age, sex, gestational age, type of cleft palate, type of syndrome, and treatment for upper airway obstruction in the neonatal period. Variables included syndromic RS (RS as part of a syndrome or RS with other associated anomalies/chromosomal defects) or isolated RS, prematurity (defined as gestational age < 37 weeks), cardiac anomalies, neurological anomalies, and surgical treatment for severe respiratory distress in the neonatal period.

The primary observational outcome measurements of this study were death and causes of death. Subsequently, associated cardiac and neurological anomalies were analyzed. All RS infants underwent a physical examination by a pediatrician. When physical examination suspected any anomalies, extensive examination was performed. Extensive cardiac examination included assessment by electrocardiography and echocardiography, and extensive neurological examination included assessment by brain magnetic resonance imaging (MRI) and echoencephalography.

Genetic workup in all infants included standard clinical examination by a geneticist, and additional testing by karyotyping and FISH for a 22q11.2 deletion. Array-CGH and next-generation sequencing were performed from 2008 and 2012, respectively, if an associated syndrome was suspected. Additionally, a recent re-evaluation of the initial genetic diagnoses was performed in our cohort [3]. We defined isolated RS in infants with a normal clinical examination, negative results from previously described tests, and a normal development. Normal development was assessed by using the Van Wiechen Scheme, which is the Dutch equivalent of the Bayley Scales of Infant Development.

Statistical analysis was performed by using the chi-square test and Fisher exact tests in IBM SPSS Statistics 24.0 (IBM Inc., NY, USA). A *p* value of < 0.05 was considered to be significant.

## Results

### Patient characteristics

At our institution, 103 consecutive infants were diagnosed with RS in the 22-year study period (1995–2016). The median follow-up period was 8.6 years (range 0.1–21.9 years). Table 1 shows the baseline characteristics of all the RS patients: isolated RS, 42%; syndromic RS, 58% (20% RS with other associated anomalies/chromosomal defects and 38% RS as part of a syndrome); median gestational age, 39.4 weeks (range 30.9–42.0 weeks); prematurity, 13%; and the presence of cleft palate, 98%. Surgical treatment for severe respiratory distress in the neonatal period was required in 35% of the infants (21 MDO’s, five TLA’s, seven tracheotomies, one MDO and later stage tracheotomy, one TLA and later stage tracheotomy, and one tracheotomy resolved by MDO).

**Table 1:** Baseline characteristics of RS infants followed at the Wilhelmina Children’s Hospital 1995–2016

Infants	Number of infants (%)	Female	Male	Gestational age (weeks)	Presence of CP (%)	CP type
Total	103 (100%)	54	49	39.4 (range 30.9–42.0)	101 (98%)	I (4); II (20); III (57); IV (20)
Isolated RS	43 (42%)	25	18	39.1 (range 32.3–42.0)	42	I (0); II (8); III (24); IV (10)
Syndromic RS	60 (58%)	29	31	38.9 (range 30.9–42.0)	59	I (4); II (12); III (33); IV (10)
RS as part of a syndrome	39 (38%)					
Stickler syndrome	16					
Treacher-Collins syndrome	2					
Spondyloepiphyseal dysplasia congenita	2					
4q deletion syndrome	1					
Auriculo-Condylar syndrome	1					
Carey-Fineman-Ziter syndrome	1					
EEC syndrome	1					
Worster-Drought syndrome	1					
Klinefelter syndrome	1					
Cerebro-costo-mandibular syndrome	1					
Catel-Manzke syndrome	1					
Yunis-Varon syndrome	1					
Van der Woude syndrome	1					
Osteopathia striata with cranial sclerosis	1					
Hyperphosphatasia mental retardation syndrome 1	1					
Hemifacial microsomia	1					
Sotos syndrome	1					
CHARGE syndrome	1					
Unknown syndrome	4					
Other associated abnormalities or chromosomal abnormalities	21 (20%)					

*RS*, Robin sequence; *Syndromic RS*, RS as part of a syndrome or RS with other associated anomalies/chromosomal defects; *CHARGE syndrome*, coloboma, heart defect, atresia choanae, retarded growth and development, genital abnormality, and ear abnormality syndrome; *EEC syndrome*, ectrodactyly ectodermal dysplasia cleft lip/palate syndrome; *CP*, cleft palate; *CP-type*, modified “Jensen et al. classification” [20]: I, submucosal cleft or bifid uvula; II, soft palate; III, soft palate and part of hard palate; IV, soft palate and hard palate up to incisive foramen

### Mortality

Ten of the 103 infants (10%) died at a median age of 0.8 years (range 0.1–5.9 years). One other infant was unvaccinated due to the parents’ religious belief and died of Haemophilus influenzae type B septic meningitis. Since this death was totally unrelated to RS, this infant was excluded from the analysis. The characteristics of the ten deceased RS infants are listed in Table 2. An even distribution of deaths was observed in our study period (1995–2016). Five females and five males died. Seven infants died of respiratory insufficiency due to various causes (two of viral pneumonia, one of aspiration pneumonia, one of pneumosepsis, two of airway obstruction problems, and one of muscle weakness). The other three infants died of arrhythmia due to hypernatremia of 167 mmol/L with urosepsis (*n* = 1), West syndrome with status epilepticus (*n* = 1), and brain ischemia after MDO surgery (*n* = 1). Nine infants had syndromic RS, and one infant had no diagnosed syndrome or other associated anomalies/chromosomal defects. This isolated RS infant died of brain ischemia due to a major complication of persistent low blood pressure during MDO surgery.

**Table 2:** Characteristics of the deceased RS infants followed at the Wilhelmina Children’s Hospital 1995–2016

Infant and year of birth	Sex	Age at death (years)	Isolated/syndromic	Syndrome	Cause of death	Cardiac-neurological examination		Surgery	Anomalies
I - 1995	F	5.9	Syndromic	Karyotype 46, XX, 8p+	Respiratory insufficiency after viral pneumonia in combination with Reye’s syndrome.	No	Yes	–	Grade IIa left ventricular bleeding, severe periventricular flaring, and dysplastic corpus callosum.
II - 1996	M	0.7	Syndromic	CHARGE syndrome	Respiratory insufficiency after viral pneumonia with CHARGE association.	Yes	Yes	–	Atrioventricular septal defect, patent ductus arteriosus, and right ventricular hypertrophy.
III - 1999	F	0.8	Syndromic	4q syndrome	Arrhythmia due to hypernatremia of 167 mmol/L and urosepsis.	Yes	Yes	TLA	Bilateral germinolytic cysts and cavum septum pellucidum. Aortic stenosis with coarctation of the aorta, multiple ventricular septal defects, and left ventricular hypertrophy.
IV - 2001	M	0.1	Syndromic	Spondyloepiphyseal dysplasia congenita syndrome.	Respiratory insufficiency after aspiration pneumonia.	No	No	–	
V - 2003	F	2.8	Syndromic	Unknown syndrome: microcephaly, blindness, severe psychomotor retardation and epilepsy.	Respiratory insufficiency after pneumosepsis, palliative treatment. History of gastroesophageal reflux with aspirations, causing recurrent airway problems.	Yes	Yes	–	Hypoplastic corpus callosum, septum pellucidum agenesis, lenticulostriatal vasculopathy, ventricular system left > right, and periventricular noduli suspected for a neuronal migration disorder.
VI - 2004	F	2.7	Syndromic	Hyperphosphatasia with mental retardation syndrome 1.	West syndrome with status epilepticus.	Yes	Yes	–	Hypoplastic corpus callosum. Ventricular septal defect.
VII - 2009	M	0.2	Syndromic	Yunis-Varon syndrome	Respiratory insufficiency after persistent upper airway obstruction that showed no improvement after TLA. Palliative treatment since persistent respiratory problems, severe dysphagia, and other complex anomalies.	Yes	Yes	TLA	Hypoplastic pons and vermis, partial agenesis of the corpus callosum Hypoplastic left ventricle complex, coarctation of the aorta, aberrant right subclavian arteries, persistent left superior vena cava, atrial septal defect, and patent ductus arteriosus.
VIII - 2010	F	0.1	Isolated	–	Post-MDO surgery severe convulsions. Brain ischemia due to low blood pressure moments during surgery and possible preoperative hypoxic moments due to RS.	Yes	Yes	MDO	
IX - 2011	M	3	Syndromic	Treacher-Collins syndrome	Respiratory insufficiency caused by upper airway obstruction (mucus), reanimation with post-anoxic brain injury and brain herniation. History of multiple hospital admissions due to aspirations and respiratory problems.	Yes	No	MDO	
X - 2013	M	0.2	Syndromic	Carey-Fineman-Ziter syndrome	Respiratory insufficiency due to muscle weakness that required persistent ventilation. Palliative treatment.	Yes	Yes	MDO	Brainstem calcifications (associated with Carey-Fineman-Ziter syndrome).

Note: 70% of all the deceased RS infants underwent both extensive cardiac and neurological examination

*M*, male; *F*, female; *RS*, Robin sequence; *Syndromic RS*, RS as part of a syndrome or RS with other associated anomalies/chromosomal defects; *MDO*, mandibular distraction osteogenesis; *TLA*, tongue-lip adhesion; *CHARGE syndrome*, coloboma, heart defect, atresia choanae, retarded growth and development, genital abnormality, and ear abnormality syndrome

### Extensive cardiac and neurological examination

In 41 infants (40%), extensive cardiac examination was performed, including 27 assessments by electrocardiography and 31 assessments by echocardiography. Extensive neurological examination was done in 42 infants (41%), of which 15 had a brain MRI and 35 an echoencephalography. The group of 41 infants that underwent extensive cardiac examination consisted of both syndromic (76%) and isolated (24%) RS infants. The 42 infants that had extensive neurological examination, also included both syndromic (69%) and isolated (31%) RS infants. When looking at the total syndromic RS group (*n* = 60), in only 52 and 48%, extensive cardiac and neurological examination was performed, respectively.

### Anomalies and risk groups

All anomalies diagnosed by extensive cardiac and neurological examination are listed in Table 3. Seventeen infants (41%) were diagnosed with cardiac anomalies, of which the ventricular septum defect (*n* = 10) was observed most frequently. Neurological anomalies were diagnosed in 15 infants (36%), and a hypoplastic corpus callosum (*n* = 5) was found most frequently. Extensive examination by electrocardiography did not reveal any anomalies.

**Table 3:** Identified anomalies of the RS infants followed at the Wilhelmina Children’s Hospital 1995–2016

Anomaly	No.
Cardiac (41% of analyzed RS infants*)	34
Ventricular septal defect	10
Patent foramen ovale	5
Patent ductus arteriosus	3
Coarctation of the aorta	2
Bicuspid aortic valve	2
Right ventricular hypertrophy	2
Atrial septal defect	1
Atrioventricular septal defect	1
Left non-compaction ventricular cardiomyopathy	1
Aberrant right subclavian arteries	1
Persistent left superior vena cava	1
Supravalvular pulmonary stenosis	1
Pulmonic stenosis	1
Left pulmonary artery stenosis	1
Left ventricular hypertrophy	1
Hypoplastic left ventricle	1
Neurologic (36% of analyzed RS infants*)	30
Hypoplastic corpus callosum	5
Cavum septum pellucidum	4
Asymmetric ventricular system	3
Hypoplastic pons	3
Bilateral germinolytic cysts	2
Hypoplastic vermis	2
Cyst	2
Grade IIa ventricular bleeding	1
Bilateral thalamic densities	1
Cavum vergae	1
Lenticulostriatal vasculopathy	1
Periventricular noduli suspected for neuronal migration disorder	1
Bilateral frontal and left periventricular aspecific white matter abnormalities	1
Typical leukomalacia abnormalities	1
Colpocephaly	1
Brainstem calcifications (associated with Carey-Fineman-Ziter syndrome)	1

*Note: some RS infants had multiple anomalies

*RS* Robin sequence

The presence of a neurological anomaly was associated with a mortality rate of 40% versus 7% in infants with no neurological anomaly (*p* = 0.016). The odds ratio for mortality was 8.3 (95% CI 1.4–49.0) for neurological anomaly versus no neurological anomaly. The mortality rate was 15% in infants with syndromic RS versus 2% in infants with isolated RS (*p* = 0.044). The other variables did not demonstrate a statistically significant association with mortality: the presence of a cardiac anomaly was associated with a mortality rate of 24% versus 17% in infants with no cardiac anomaly (*p* = 0.698), surgical treatment for severe respiratory distress with 14% versus 8% for noninvasive treatment (*p* = 0.318), and premature birth with 2% versus 8% for full-term birth (*p* = 0.621).

## Discussion

This retrospective study of a large cohort of RS infants provides new insight into the mortality of RS and the associated risk factors. We report a mortality rate of 10% in RS infants, and mortality significantly associated with the presence of neurological anomalies and with the diagnosis of syndromic RS. Mortality was not significantly associated with the presence of a cardiac anomaly, surgical treatment for severe respiratory distress in the neonatal period, or prematurity.

Our reported mortality rate is in line with the previously described mortality rates in RS infants, which range from 2 to 26% [9, 11, 12, 14, 15, 19, 21, 29, 32, 33, 35, 37], although it was higher than what we expected when the study was initiated. Our group of deceased infants consists of a highly heterogeneous group (Table 2). Costa et al. reported in their cohort of 181 RS infants (the largest cohort available) a higher mortality rate of 17%, and in their series, only syndromic RS infants died (*p* = 0.002) [12]. In our cohort, nine syndromic RS infants and one isolated RS infant died, and we observed a significant association between syndromic RS and mortality (*p* = 0.044). The death of this isolated RS infant should be discussed. Sadly, this infant developed severe convulsions post-MDO surgery, and a CT scan of the brain demonstrated severe lesions of ischemia. The brain ischemia was interpreted by the low blood pressure moments during MDO surgery in combination with the preoperative hypoxic moments due to RS. This emphasizes the fragility of RS in relationship to anesthesia and surgical interventions. Moreover, a complete genetic workup was not made for this infant, and it is possible that, with time, these genetic investigations could have revealed a possible genetic cause or syndrome. Furthermore, a recent study by Basart et al. emphasized the importance of repeated genetic evaluation. After re-evaluation, 25% of patients had a new genetic diagnosis [3]. Subsequently, with a more universally accepted minimum “norm” of gene analysis performed by the clinical geneticist, especially since the introduction of the next-generation sequencing, more infants could be diagnosed with an additional genetic condition [7].

In our heterogeneous group of deceased infants, we could identify seven infants that died of respiratory insufficiency due to different causes (two of viral pneumonia, one of aspiration pneumonia, one of pneumosepsis, two of airway obstruction problems, and one of muscle weakness). All these seven infants had syndromic RS, and a wide range of age-at-death was observed (0.1–5.9 years). This indicates that clinicians should be more aware of respiratory problems in syndromic RS infants, also after the first year of life. This is in line with Van Lieshout et al., who reported that, between the age of 1 and 18 years, almost one out of four RS infants continues to have respiratory problems. Additionally, RS infants who need respiratory support early after birth are at risk of continuing or re-developing obstructive sleep apnea after the age of 1 year [34]. In our study, we could relate the cause of respiratory insufficiency to upper airway obstruction in only two infants (VII and IX). In the other infants (I, V, X), the respiratory distress might be related to a neurological cause, based on the presence of their neurological anomalies. This might result in pharyngo-laryngeal dyscoordination that could predispose these infants to the risk of respiratory insufficiency.

This study has several limitations that should be discussed. First, we experienced an important variability in follow-up time ranging from 0.1 to 21.9 years, with a median of 8.6 years. The lower range of our follow-up time is explained by the RS infants in our cohort that died at a very young age.

Second, the present study only identified two RS infants without the presence of a cleft palate. The recent international consensus on the diagnosis of RS states that cleft palate is not mandatory for the diagnosis of RS, although it is present in about 90% of RS infants [7]. However, a report in 2009 demonstrated that there was no uniformity among clinicians in the Netherlands involved in craniofacial care in defining RS and the inclusion of cleft palate as part of the sequence [5]. It is possible that, in our study period, infants without the presence of cleft palate were not identified as RS at our institution. This would explain the high incidence of cleft palate (98%) in our RS cohort.

Third, having a neurological anomaly and an associated syndrome might be confounding variables. In the future, larger RS cohorts are necessary to make a distinction between these variables.

Lastly, not all infants had the same cardiac and neurological workup; this is because extensive cardiac and neurological examination was only performed, when physical examination suspected any anomalies. This diagnostic workup remained unchanged over the study period and resulted in extensive cardiac and neurological examination of 40% and 41% of our infants, respectively. Our findings of 41% cardiac and 36% neurological anomalies are higher compared to other studies [12, 23, 26, 28, 37]. However, the criteria for performing extensive cardiac or neurological examination in these studies were not specified. Previously, reported cardiac anomalies in RS infants range from 7 to 31%, and neurologic anomalies were observed in 25% [12, 23, 26, 28, 37]. Extensive examination was performed in only a subgroup of our RS infants, which was suspected for anomalies after physical examination; these infants were also more likely to have anomalies, which could explain our higher incidence of anomalies. On the other hand, we cannot exclude all cardiac and neurological anomalies in our cohort since, of the syndromic RS infants, only 52 and 48% had extensive cardiac and neurological examinations, respectively. By analyzing all of the different anomalies, we could only identify the ventricular septum defect and the hypoplastic corpus callosum as frequently associated anomalies in RS. The other identified anomalies were diverse and indicated the heterogeneity of RS.

However, in our institution, physical examination combined with extensive neurological examination could identify a group of RS infants that had increased mortality, 40% in RS infants with a neurological anomaly (*p* = 0.016). This is in line with the findings of Costa et al. who reported cardiac and neurological anomalies significantly associated with an increased mortality rate [11]. Interestingly, extensive cardiac and neurological examination was not only performed in the syndromic RS infants. The pediatrician’s physical examination resulted in extensive cardiac and neurological examination in 24% and 31% of the isolated RS infants. The demonstrated significant association between the presence of neurological anomalies and an increased mortality rate advocates that all RS infants should be investigated for the presence of anomalies.

## Conclusion

RS infants represent a heterogeneous population and are associated with a high level of underlying syndromes. The present study reports a mortality rate of 10%, which was significantly associated with syndromic RS and the presence of neurological anomalies. A multidisciplinary approach in all infants born with RS, including genetic testing and examination of neurological anomalies in a standardized way, is crucial to identify infants with underlying syndromes potentially associated with increased mortality. We suggest future prospective multicenter studies that extensively examine the possible genetic diagnosis and congenital anomalies in a standardized way in infants with RS.

## Abbreviations

MDO: Mandibular distraction osteogenesis
MRI: Magnetic resonance imaging
RS: Robin sequence
TLA: Tongue-lip adhesion
